# (*E*)-3-(Furan-2-yl)-1-(4-meth­oxy­phen­yl)prop-2-en-1-one

**DOI:** 10.1107/S160053681104373X

**Published:** 2011-11-05

**Authors:** Kamini Kapoor, Vivek K. Gupta, Rajni Kant, Jalpa R. Pandya, Sunil B. Lade, Hitendra S. Joshi

**Affiliations:** a X-ray Crystallography Laboratory, Post-Graduate Department of Physics and Electronics, University of Jammu, Jammu Tawi 180 006, India; b Chemistry Department, Saurashtra University, Rajkot 360 005, India

## Abstract

In the title mol­ecule, C_14_H_12_O_3_, the prop-2-en-1-one unit forms dihedral angles of 12.96 (5) and 7.89 (7)° with the 4-meth­oxy­phenyl group and the furan ring, respectively. The furan and benzene rings form a dihedral angle of 8.56 (5)°. In the crystal, C—H⋯π and π–π inter­actions are observed between the benzene and heterocyclic rings [centroid–centroid distance = 3.760 (1) Å].

## Related literature

For biological properties of chalcone derivatives, see: Hsieh *et al.* (1998 ▶); Anto *et al.* (1994 ▶); Bhat *et al.* (2005 ▶); Xue *et al.* (2004 ▶). For the effectiveness of chalcones against cancer, see: De Vincenzo *et al.* (2000 ▶); Dimmock *et al.* (1998 ▶). For related structures, see: Fun *et al.* (2008 ▶); Guo *et al.* (2008 ▶).
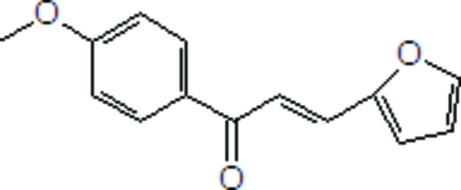


## Experimental

### 

#### Crystal data

C_14_H_12_O_3_
*M* *_r_* = 228.24
Monoclinic, 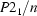

*a* = 7.1583 (3) Å
*b* = 19.1516 (8) Å
*c* = 8.4293 (3) Å
β = 94.357 (4)°
*V* = 1152.26 (8) Å^3^
*Z* = 4
Mo *K*α radiation
μ = 0.09 mm^−1^
*T* = 293 K
0.3 × 0.2 × 0.2 mm

#### Data collection

Oxford Diffraction Xcalibur S diffractometer
Absorption correction: multi-scan (*CrysAlis PRO*; Oxford Diffraction, 2007 ▶) *T*_min_ = 0.976, *T*_max_ = 1.000
13386 measured reflections
2027 independent reflections
1546 reflections with *I* > 2σ(*I*)
*R*_int_ = 0.028

#### Refinement

*R*[*F* ^2^ > 2σ(*F* ^2^)] = 0.039
*wR*(*F* ^2^) = 0.096
*S* = 1.03
2027 reflections
156 parameters
H-atom parameters constrained
Δρ_max_ = 0.13 e Å^−3^
Δρ_min_ = −0.13 e Å^−3^

### 

Data collection: *CrysAlis PRO* (Oxford Diffraction, 2007 ▶); cell refinement: *CrysAlis PRO*; data reduction: *CrysAlis PRO*; program(s) used to solve structure: *SHELXS97* (Sheldrick, 2008 ▶); program(s) used to refine structure: *SHELXL97* (Sheldrick, 2008 ▶); molecular graphics: *ORTEP-3* (Farrugia, 1997 ▶); software used to prepare material for publication: *PLATON* (Spek, 2009 ▶) and *PARST* (Nardelli, 1995 ▶).

## Supplementary Material

Crystal structure: contains datablock(s) I, global. DOI: 10.1107/S160053681104373X/gk2411sup1.cif

Structure factors: contains datablock(s) I. DOI: 10.1107/S160053681104373X/gk2411Isup2.hkl

Supplementary material file. DOI: 10.1107/S160053681104373X/gk2411Isup3.cml

Additional supplementary materials:  crystallographic information; 3D view; checkCIF report

## Figures and Tables

**Table 1:** Hydrogen-bond geometry (Å, °) *Cg*1 is the centroid of the furan ring.

*D*—H⋯*A*	*D*—H	H⋯*A*	*D*⋯*A*	*D*—H⋯*A*
C10—H10⋯*Cg*1^i^	0.93	2.76	3.592 (2)	149

Symmetry code: (i) 
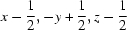
.

**Fig. 1.**
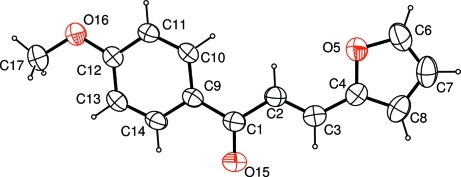
ORTEP view of the title molecule with displacement ellipsoids drawn at the 40% probability level. H atoms are shown as small spheres of arbitrary radii.

**Fig. 2.**
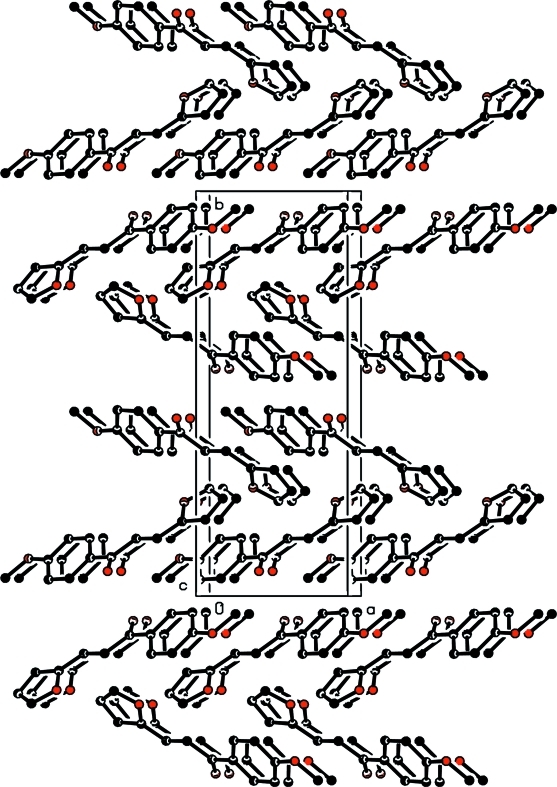
The packing arrangement of molecules viewed down the c axis.

## supplementary crystallographic information

### Comment

Chalcones and its derivatives have attracted particular interest during the last few decades due to use of such system as the core structure in many drug substances covering wide range of pharmacological application. Chalcone derivatives are reported to possess a broad spectrum of biological properties (Bhat *et al.*, 2005; Xue *et al.*, 2004; Hsieh *et al.*,1998; Anto *et al.*, 1994; De Vincenzo *et al.*,2000; Dimmock *et al.*, 1998). The bond lengths and angles observed in (I) show normal values and are comparable with related structures (Fun *et al.*, 2008; Guo *et al.*, 2008). In (I), the molecule exhibits an E configuration with respect to the C2=C3 double bond with the C1—C2—C3—C4 torsion angle being 179.95 (15)°. The least-square plane through the enone moiety (O15C1C2C3) makes dihedral angles of 12.96 (5)° and 7.89 (7)° with the benzene and furan rings, respectively. The dihedral angle between the 4-methoxy-phenyl group and furan ring is 8.56 (5)°, indicating that they are slightly twisted reletive to each other. While no classical hydrogen bonds are present, the C—H···π hydrogen bonds (*Cg*1 is the centroid of the furan ring and *Cg*2 is the centroid of the benzene ring are stabilizing the crystal structure. The crystal structure is further stabilized by π-π interactions between the benzene ring at (*x*, *y*, *z*)and furan ring at (1 + *x*, *y*, *z*) [centroid separation = 3.760 (1) Å, interplanar spacing = 3.510 Å and centroid shift = 1.35 Å].

### Experimental

A mixture of the *p*-methoxyacetophenone (1.5 g, 0.01 mol), furfural (0.9 ml, 0.01 mol) and 40% NaOH (1 ml) was stirred in methanol (8 ml) for 24 h to afford the title compound (m.p. 341 K). Single crystals suitable for X-ray measurements were obtained by recrystallization from methanol at room temperature.

### Refinement

All H atoms were positioned geometrically and treated as riding atoms [C—H = 0.93–0.96 Å].

### Crystal data

**Table:**

C_14_H_12_O_3_	*F*(000) = 480
*M**_r_* = 228.24	*D*_x_ = 1.316 Mg m^−^^3^
Monoclinic, *P*2_1_/*n*	Melting point: 341 K
Hall symbol: -P 2yn	Mo *K*α radiation, λ = 0.71073 Å
*a* = 7.1583 (3) Å	Cell parameters from 5366 reflections
*b* = 19.1516 (8) Å	θ = 3.6–29.0°
*c* = 8.4293 (3) Å	µ = 0.09 mm^−^^1^
β = 94.357 (4)°	*T* = 293 K
*V* = 1152.26 (8) Å^3^	Block, yellow
*Z* = 4	0.3 × 0.2 × 0.2 mm

### Data collection

**Table:**

Oxford Diffraction Xcalibur S diffractometer	2027 independent reflections
Radiation source: fine-focus sealed tube	1546 reflections with *I* > 2σ(*I*)
graphite	*R*_int_ = 0.028
Detector resolution: 16.1049 pixels mm^-1^	θ_max_ = 25.0°, θ_min_ = 3.6°
ω scans	*h* = −8→8
Absorption correction: multi-scan (*CrysAlis PRO*; Oxford Diffraction, 2007)	*k* = −22→22
*T*_min_ = 0.976, *T*_max_ = 1.000	*l* = −10→10
13386 measured reflections	

### Refinement

**Table:**

Refinement on *F*^2^	Secondary atom site location: difference Fourier map
Least-squares matrix: full	Hydrogen site location: inferred from neighbouring sites
*R*[*F*^2^ > 2σ(*F*^2^)] = 0.039	H-atom parameters constrained
*wR*(*F*^2^) = 0.096	*w* = 1/[σ^2^(*F*_o_^2^) + (0.0349*P*)^2^ + 0.2369*P*] where *P* = (*F*_o_^2^ + 2*F*_c_^2^)/3
*S* = 1.03	(Δ/σ)_max_ < 0.001
2027 reflections	Δρ_max_ = 0.13 e Å^−^^3^
156 parameters	Δρ_min_ = −0.13 e Å^−^^3^
0 restraints	Extinction correction: *SHELXL97* (Sheldrick, 2008), Fc^*^=kFc[1+0.001xFc^2^λ^3^/sin(2θ)]^-1/4^
Primary atom site location: structure-invariant direct methods	Extinction coefficient: 0.0089 (17)

### Special details

**Table:**

Geometry. All e.s.d.'s (except the e.s.d. in the dihedral angle between two l.s. planes) are estimated using the full covariance matrix. The cell e.s.d.'s are taken into account individually in the estimation of e.s.d.'s in distances, angles and torsion angles; correlations between e.s.d.'s in cell parameters are only used when they are defined by crystal symmetry. An approximate (isotropic) treatment of cell e.s.d.'s is used for estimating e.s.d.'s involving l.s. planes.
Refinement. Refinement of *F*^2^ against ALL reflections. The weighted *R*-factor *wR* and goodness of fit *S* are based on *F*^2^, conventional *R*-factors *R* are based on *F*, with *F* set to zero for negative *F*^2^. The threshold expression of *F*^2^ > σ(*F*^2^) is used only for calculating *R*-factors(gt) *etc*. and is not relevant to the choice of reflections for refinement. *R*-factors based on *F*^2^ are statistically about twice as large as those based on *F*, and *R*- factors based on ALL data will be even larger.

### Fractional atomic coordinates and isotropic or equivalent isotropic displacement parameters (Å^2^)

**Table:**

	*x*	*y*	*z*	*U*_iso_*/*U*_eq_	
C1	0.5604 (2)	0.90359 (8)	−0.00421 (19)	0.0538 (4)	
C2	0.3963 (2)	0.85830 (8)	0.01140 (19)	0.0553 (4)	
H2	0.3954	0.8290	0.0993	0.066*	
C3	0.2496 (2)	0.85775 (8)	−0.09527 (19)	0.0548 (4)	
H3	0.2546	0.8877	−0.1818	0.066*	
C4	0.0853 (2)	0.81579 (8)	−0.09015 (19)	0.0543 (4)	
C6	−0.0871 (3)	0.73468 (11)	0.0068 (2)	0.0783 (6)	
H6	−0.1271	0.6994	0.0720	0.094*	
C7	−0.1863 (3)	0.75916 (11)	−0.1198 (2)	0.0774 (6)	
H7	−0.3048	0.7446	−0.1591	0.093*	
C8	−0.0767 (2)	0.81144 (10)	−0.1830 (2)	0.0701 (5)	
H8	−0.1095	0.8383	−0.2727	0.084*	
C9	0.7104 (2)	0.90560 (8)	0.12703 (18)	0.0481 (4)	
C10	0.7260 (2)	0.85704 (8)	0.24994 (19)	0.0545 (4)	
H10	0.6348	0.8227	0.2552	0.065*	
C11	0.8739 (2)	0.85887 (8)	0.3638 (2)	0.0581 (4)	
H11	0.8827	0.8254	0.4440	0.070*	
C12	1.0100 (2)	0.91016 (8)	0.36004 (19)	0.0519 (4)	
C13	0.9963 (2)	0.95944 (8)	0.2403 (2)	0.0582 (4)	
H13	1.0863	0.9943	0.2365	0.070*	
C14	0.8480 (2)	0.95650 (8)	0.1263 (2)	0.0571 (4)	
H14	0.8399	0.9899	0.0460	0.068*	
C17	1.3024 (2)	0.95549 (10)	0.4718 (2)	0.0740 (5)	
H17A	1.3600	0.9490	0.3737	0.111*	
H17B	1.3931	0.9471	0.5596	0.111*	
H17C	1.2567	1.0025	0.4773	0.111*	
O5	0.08105 (16)	0.76778 (6)	0.02948 (13)	0.0675 (4)	
O15	0.57315 (17)	0.93872 (7)	−0.12483 (15)	0.0750 (4)	
O16	1.14981 (16)	0.90766 (6)	0.47907 (14)	0.0687 (4)	

### Atomic displacement parameters (Å^2^)

**Table:**

	*U*^11^	*U*^22^	*U*^33^	*U*^12^	*U*^13^	*U*^23^
C1	0.0570 (10)	0.0505 (9)	0.0550 (10)	0.0044 (8)	0.0116 (8)	0.0056 (8)
C2	0.0572 (10)	0.0542 (10)	0.0547 (10)	−0.0003 (8)	0.0049 (8)	0.0055 (8)
C3	0.0612 (10)	0.0509 (9)	0.0527 (9)	0.0065 (8)	0.0076 (8)	−0.0005 (8)
C4	0.0568 (10)	0.0557 (10)	0.0503 (9)	0.0069 (8)	0.0032 (7)	−0.0081 (8)
C6	0.0736 (13)	0.0847 (14)	0.0782 (13)	−0.0259 (11)	0.0153 (10)	−0.0189 (11)
C7	0.0565 (11)	0.0969 (15)	0.0789 (14)	−0.0069 (11)	0.0047 (10)	−0.0358 (12)
C8	0.0661 (12)	0.0820 (13)	0.0605 (11)	0.0119 (10)	−0.0062 (9)	−0.0151 (10)
C9	0.0499 (9)	0.0438 (8)	0.0519 (9)	0.0006 (7)	0.0129 (7)	0.0024 (7)
C10	0.0534 (9)	0.0495 (9)	0.0609 (10)	−0.0112 (7)	0.0077 (8)	0.0065 (8)
C11	0.0611 (10)	0.0533 (10)	0.0596 (10)	−0.0100 (8)	0.0024 (8)	0.0121 (8)
C12	0.0513 (9)	0.0496 (9)	0.0554 (10)	−0.0028 (7)	0.0089 (7)	−0.0027 (8)
C13	0.0574 (10)	0.0506 (10)	0.0675 (11)	−0.0128 (8)	0.0112 (8)	0.0023 (8)
C14	0.0622 (10)	0.0494 (9)	0.0609 (10)	−0.0050 (8)	0.0126 (8)	0.0119 (8)
C17	0.0587 (11)	0.0759 (12)	0.0871 (14)	−0.0184 (9)	0.0024 (9)	−0.0030 (11)
O5	0.0650 (8)	0.0726 (8)	0.0642 (8)	−0.0123 (6)	−0.0009 (6)	−0.0002 (6)
O15	0.0731 (8)	0.0854 (9)	0.0663 (8)	−0.0068 (7)	0.0035 (6)	0.0267 (7)
O16	0.0625 (7)	0.0702 (8)	0.0717 (8)	−0.0168 (6)	−0.0055 (6)	0.0065 (6)

### Geometric parameters (Å, °)

**Table:**

C1—O15	1.2284 (18)	C9—C14	1.386 (2)
C1—C2	1.474 (2)	C9—C10	1.390 (2)
C1—C9	1.483 (2)	C10—C11	1.374 (2)
C2—C3	1.330 (2)	C10—H10	0.9300
C2—H2	0.9300	C11—C12	1.385 (2)
C3—C4	1.428 (2)	C11—H11	0.9300
C3—H3	0.9300	C12—O16	1.3633 (18)
C4—C8	1.351 (2)	C12—C13	1.380 (2)
C4—O5	1.3667 (19)	C13—C14	1.378 (2)
C6—C7	1.322 (3)	C13—H13	0.9300
C6—O5	1.361 (2)	C14—H14	0.9300
C6—H6	0.9300	C17—O16	1.4307 (19)
C7—C8	1.402 (3)	C17—H17A	0.9600
C7—H7	0.9300	C17—H17B	0.9600
C8—H8	0.9300	C17—H17C	0.9600
			
O15—C1—C2	120.42 (15)	C11—C10—C9	121.16 (14)
O15—C1—C9	120.53 (15)	C11—C10—H10	119.4
C2—C1—C9	119.05 (14)	C9—C10—H10	119.4
C3—C2—C1	122.54 (15)	C10—C11—C12	120.51 (15)
C3—C2—H2	118.7	C10—C11—H11	119.7
C1—C2—H2	118.7	C12—C11—H11	119.7
C2—C3—C4	126.45 (15)	O16—C12—C13	124.72 (14)
C2—C3—H3	116.8	O16—C12—C11	115.88 (14)
C4—C3—H3	116.8	C13—C12—C11	119.40 (15)
C8—C4—O5	108.66 (15)	C14—C13—C12	119.35 (15)
C8—C4—C3	133.60 (17)	C14—C13—H13	120.3
O5—C4—C3	117.73 (13)	C12—C13—H13	120.3
C7—C6—O5	111.29 (18)	C13—C14—C9	122.40 (15)
C7—C6—H6	124.4	C13—C14—H14	118.8
O5—C6—H6	124.4	C9—C14—H14	118.8
C6—C7—C8	106.16 (17)	O16—C17—H17A	109.5
C6—C7—H7	126.9	O16—C17—H17B	109.5
C8—C7—H7	126.9	H17A—C17—H17B	109.5
C4—C8—C7	107.71 (18)	O16—C17—H17C	109.5
C4—C8—H8	126.1	H17A—C17—H17C	109.5
C7—C8—H8	126.1	H17B—C17—H17C	109.5
C14—C9—C10	117.17 (15)	C6—O5—C4	106.19 (14)
C14—C9—C1	119.32 (14)	C12—O16—C17	117.81 (13)
C10—C9—C1	123.45 (14)		
			
O15—C1—C2—C3	−5.9 (2)	C1—C9—C10—C11	176.21 (15)
C9—C1—C2—C3	174.64 (15)	C9—C10—C11—C12	1.1 (3)
C1—C2—C3—C4	179.95 (14)	C10—C11—C12—O16	179.49 (15)
C2—C3—C4—C8	176.35 (18)	C10—C11—C12—C13	−0.3 (2)
C2—C3—C4—O5	−4.8 (2)	O16—C12—C13—C14	179.98 (15)
O5—C6—C7—C8	−0.2 (2)	C11—C12—C13—C14	−0.3 (2)
O5—C4—C8—C7	0.07 (19)	C12—C13—C14—C9	0.1 (2)
C3—C4—C8—C7	179.02 (17)	C10—C9—C14—C13	0.7 (2)
C6—C7—C8—C4	0.1 (2)	C1—C9—C14—C13	−176.87 (15)
O15—C1—C9—C14	11.8 (2)	C7—C6—O5—C4	0.3 (2)
C2—C1—C9—C14	−168.82 (13)	C8—C4—O5—C6	−0.21 (18)
O15—C1—C9—C10	−165.65 (15)	C3—C4—O5—C6	−179.35 (14)
C2—C1—C9—C10	13.8 (2)	C13—C12—O16—C17	−6.0 (2)
C14—C9—C10—C11	−1.2 (2)	C11—C12—O16—C17	174.30 (14)

### Hydrogen-bond geometry (Å, °)

**Table:**

*D*—H···*A*	*D*—H	H···*A*	*D*···*A*	*D*—H···*A*
C10—H10···Cg1^i^	0.93	2.76	3.592 (2)	149
C17—H17C···Cg2^ii^	0.96	3.07	3.788 (2)	132

Symmetry codes: (i) *x*−1/2, −*y*+1/2, *z*−1/2; (ii) −*x*, −*y*, −*z*+1.

## References

[bb1] Anto, R. J., Kuttan, G., Kuttan, R., Sathyanarayana, K. & Rao, M. N. A. (1994). *J. Clin. Biochem. Nutr.* **17**, 73–80.

[bb2] Bhat, B. A., Dhar, K. L., Puri, S. C., Saxena, A. K., Shanmugavel, M. & Qazi, G. N. (2005). *Bioorg. Med. Chem. Lett.* **15**, 3177–3180. 10.1016/j.bmcl.2005.03.121 15893928

[bb3] De Vincenzo, R., Ferlini, C., Distefano, M., Gaggini, C., Riva, A., Bombardelli, E., Morazzoni, P., Valenti, P., Belluti, F., Ranelletti, F. O., Mancuso, S. & Scambia, G. (2000). *Cancer Chemother. Pharmacol.* **46**, 305–312. 10.1007/s002800000160 11052628

[bb4] Dimmock, J. R., *et al.* (1998). *J. Med. Chem.* **41**, 1014–1026. 10.1021/jm970432t 9544201

[bb5] Farrugia, L. J. (1997). *J. Appl. Cryst.* **30**, 565.

[bb6] Fun, H.-K., Patil, P. S., Jebas, S. R. & Dharmaprakash, S. M. (2008). *Acta Cryst.* E**64**, o1467. 10.1107/S1600536808020965 PMC2962097 21203181

[bb7] Guo, H.-M., Wang, X.-B. & Jian, F.-F. (2008). *Acta Cryst.* E**64**, o1951. 10.1107/S1600536808029152 PMC2959475 21201155

[bb8] Hsieh, H. K., Lee, T. H., Wang, J. P., Wang, J. J. & Lin, C. N. (1998). *Pharm. Res.* **15**, 39–46. 10.1023/a:1011940401754 9487544

[bb9] Nardelli, M. (1995). *J. Appl. Cryst.* **28**, 659.

[bb10] Oxford Diffraction (2007). *CrysAlis PRO* Oxford Diffraction Ltd, Abingdon, Oxfordshire, England.

[bb11] Sheldrick, G. M. (2008). *Acta Cryst.* A**64**, 112–122. 10.1107/S0108767307043930 18156677

[bb12] Spek, A. L. (2009). *Acta Cryst.* D**65**, 148–155. 10.1107/S090744490804362X PMC2631630 19171970

[bb13] Xue, C. X., Cui, S. Y., Liu, M. C., Hu, Z. D. & Fan, B. T. (2004). *Eur. J. Med. Chem.* **39**, 745–753. 10.1016/j.ejmech.2004.05.009 15337287

